# Overexpressing of *OsAMT1-3*, a High Affinity Ammonium Transporter Gene, Modifies Rice Growth and Carbon-Nitrogen Metabolic Status

**DOI:** 10.3390/ijms16059037

**Published:** 2015-04-23

**Authors:** Aili Bao, Zhijun Liang, Zhuqing Zhao, Hongmei Cai

**Affiliations:** Key Laboratory of Arable Land Conservation (Middle and Lower Reaches of Yangtze River), Microelement Research Center, Ministry of Agriculture, Huazhong Agricultural University, Wuhan 430070, China; E-Mails: baoaili19870212@163.com (A.B.); zhijun.l@webmail.hzau.edu.cn (Z.L.); zzq@mail.hzau.edu.cn (Z.Z.)

**Keywords:** *AMT1-3*, carbon-nitrogen metabolism, ^15^N tracer, overexpressing, rice

## Abstract

*AMT1-3* encodes the high affinity NH_4_^+^ transporter in rice roots and is predominantly expressed under nitrogen starvation. In order to evaluate the effect of *AMT1-3* gene on rice growth, nitrogen absorption and metabolism, we generated *AMT1-3*-overexpressing plants and analyzed the growth phenotype, yield, carbon and nitrogen metabolic status, and gene expression profiles. Although *AMT1-3* mRNA accumulated in transgenic plants, these plants displayed significant decreases in growth when compared to the wild-type plants. The nitrogen uptake assay using a ^15^N tracer revealed poor nitrogen uptake ability in *AMT1-3*-overexpressing plants. We found significant decreases in *AMT1-3*-overexpressing plant leaf carbon and nitrogen content accompanied with a higher leaf C/N ratio. Significant changes in soluble proteins and carbohydrates were also observed in *AMT1-3*-overexpressing plants. In addition, metabolite profile analysis demonstrated significant changes in individual sugars, organic acids and free amino acids. Gene expression analysis revealed distinct expression patterns of genes that participate in carbon and nitrogen metabolism. Additionally, the correlation between the metabolites and gene expression patterns was consistent in *AMT1-3*-overexpressing plants under both low and high nitrogen growth conditions. Therefore, we hypothesized that the carbon and nitrogen metabolic imbalance caused by *AMT1-3* overexpressing attributed to the poor growth and yield of transgenic plants.

## 1. Introduction

Nitrogen (N) is one of the essential macronutrients required for plant growth and development and thus is often a major limiting factor for plant productivity and crop yield [1,2]. Nitrogen is not only a constituent of key cell molecules, such as amino acids, nucleic acids, chlorophyll, ATP and several plant hormones, but is also the pivotal regulator of numerous biological processes, including carbon metabolism, amino acid metabolism and protein synthesis [3,4]. Higher plants can absorb and use various forms of nitrogen components from the soil, most notably the inorganic ions ammonium (NH_4_^+^) and nitrate (NO_3_^−^). These ions are believed to be the principal nitrogen sources for plant growth in agricultural and most natural environments, as it is required in greater amounts than any other mineral nutrient [5]. Because ammonium assimilation requires less energy than nitrate, ammonium is the preferential nitrogen source for root uptake, particularly in nitrogen-deficient plants [6,7]. Carbon (C) is also essential for plants to keep their fundamental growth and development. Various carbohydrates, for example, glucose, sucrose and organic acids provide both the energy and the carbon skeletons for NH_4_^+^ assimilation to produce amino acids and proteins. The proteins, in particular enzymes, are essential for nearly all cellular activities. Several studies have emphasized the importance of the coordination of carbon and nitrogen metabolism [8,9,10,11,12]. The optimal functioning of the metabolic pathways for carbon and nitrogen assimilation in plants, and maintaining an appropriate balance or ratio of carbohydrates to nitrogen metabolites in the cell, which is referred to as the “carbon/nitrogen balance”, are critical for determining plant growth, biomass accumulation and seed production [8,9,10,11,12].

Rice (*Oryza sativa*), a staple food for over half of the world’s population, is one of the most important crops worldwide. NH_4_^+^ is the major available form of nitrogen that is used for growing rice plants in paddy fields and requires ammonium transporters (AMTs) in the root plasma membrane, which belong to the AMT/MEP/Rh (Ammonium Transporter/Methylamine Permease/Rhesus) protein superfamily with homologs in bacteria and fungi [13,14]. Several ammonia transporters, including bacterial AMT; plant AMT; and human RhAG, RhBG, and RhCG, have been suggested to support electroneutral NH_3_ transport, NH_3_/H^+^ symport, NH_4_^+^ transport, NH_4_^+^/H^+^ antiport and NH_4_^+^/H^+^ symport [15,16,17,18,19,20,21,22,23,24,25]. AMT/MEP proteins are integral membrane proteins that harbor 11 transmembrane helices and intracellular *C* terminals [26,27]. Biochemical studies have revealed that plant AMTs form trimers [16]. Recently, a different membrane protein type, aquaporins from the MIP (Major Intrinsic Proteins) superfamily, was also demonstrated to transport ammonia [28].

The cloning and expression analysis of *AMT* genes in several plant species have been reported to date. *Arabidopsis* harbors six AMT-type ammonium transporters, which are encoded by five genes (*AtAMT1-1*, *AtAMT1-*2, *AtAMT1-3*, *AtAMT1-5*, *AtAMT2-1*) expressed in the roots and one gene (*AtAMT1-4*) expressed in the pollen [29]. *AtAMT1-1* and *AtAMT1-3* are expressed in rhizodermal and cortical cells, where they confer high-capacity and high-affinity ammonium uptake [30,31]. In addition, reciprocal leaf and root expression of *AtAMT1-1* in response to nitrogen starvation has been reported by Engineer and Kranz [32]. *AtAMT1-2* is expressed in endodermal and cortical cells and most likely plays a major role in the uptake and retrieval of ammonium from the apoplast in the root with a lower-affinity [31]. However, *AtAMT2-1* is more highly expressed in shoots relative to roots, and no ammonium influx activity has been observed in the root [31,33,34]. *AtAMT1-5* is expressed in rhizodermal root cells and contributes 5%–10% to the overall ammonium uptake capacity [31]. *AtAMT1-4* is a pollen-specific high-affinity ammonium transporter in the plasma membrane [29]. Rice roots harbor four families of *AMT* genes: *OsAMT1*, *OsAMT2*, *OsAMT3* and *OsAMT4*. *OsAMT1* members have been characterized as a high-affinity transport system (HAT), share a highly similar sequence to each other, while the other three families have been characterized as a low-affinity transport system (LAT) [13,35]. Sonoda *et al.* [35] demonstrated distinct expression patterns in the *OsAMT1* gene family. *OsAMT1-1* is constitutively expressed in the shoots but is stimulated by ammonium in the roots; *OsAMT1-2* expression is root-specific and ammonium-inducible, whereas *OsAMT1-3* is also expressed specifically in the roots but is repressed by nitrogen. *OsAMT2-1* encodes an ammonium transporter, which is more closely related to the yeast MEP transporter sequence and is constitutively expressed in the shoots and roots [36,37]. Several studies revealed a feedback regulation and a distinct nitrogen-dependent regulation for the rice *AMT* genes, which differs from that in tomato or *Arabidopsis*. Sonoda *et al.* identified cytosolic glutamine as a promising regulatory factor of the *OsAMT1* genes [35]. Additionally, *AMT* genes in several other plant species have been identified and characterized, including *Brassica napus* (*BnAMT1-2*) [38], *Lotus japonicas* (*LjAMT1-1*, *LjAMT2-1*) [39,40] and *Lycopersicon esculentum* (*LeAMT1-1*, *LeAMT1-2* and *LeAMT1-3*) [41,42,43].

Generally, the expression of the AMT1 isolated from different plant species are transcriptionally regulated. However, ectopically expressing of the *AtAMT1-1* gene under the control of a 35S promoter in transgenic tobacco plants revealed that the *AtAMT1-1* gene can also be post-transcriptionally regulated [44]. A post-translational regulation of ammonium transport activity has also been described for the AMT1 proteins in *Arabidopsis*. Loqué *et al.* reported that the cytosolic trans-activation domain in AMT was essential for ammonium uptake, a mutation in the cytosolic AMT1-1 *C* terminus attenuated the strict dependence on allosteric trans-activation [45,46]. The oligomerization of plant AMTs is critical for allosteric regulation of transport activity, in which the conserved cytosolic *C* terminus functions as a trans-activator [47]. AMT1-1 and AMT1-2 are allosterically regulated by *C*-terminal phosphorylation, which trans-inhibited the activation of AMT1 subunits in a trimeric complex [45,48,49]. AMT1-3 harbors a phosphomimic residue in its *C* terminus regulates both homo- and hetero-trimers in a dominant-negative fashion *in vivo* [47]. *C*-terminal phosphorylation of AMT1 is rapidly triggered by an external ammonium supply and decreases ammonium uptake by the roots and thereby quickly inactivating transport in a potentially toxic environment [49]. Additionally, Graff *et al.* [50] also reported that *N-*terminal cysteines affected the oligomer stability of the allosterically regulated ammonium transporter LeAMT1-1.

As we known, in agriculture, rice growth and yield requires abundant N. In order to meet the high production of rice, large amounts of synthetic N fertilizers are applied on arable land by farmers. However, crop plants use less than half of the applied N fertilizers [51]. Zhang *et al.* [52] reported that the N use efficiency of midseason rice in China is less than 30%. The applications of large quantities of synthetic N fertilizers to increase crop yield are not economically sustainable and placed a heavy economic burden on farmers, and also result in environmental pollutions. Because of the high-affinity NH_4_^+^ uptake function of *AMT1-3* in rice, it is a good candidate gene for use in transformation strategies aimed at improving nitrogen use efficiency and rice yield. However, no study has been reported about the function of *AMT1-3* gene in rice growth and carbon-nitrogen metabolism to date. In order to evaluate the effect of *AMT1-3* gene on rice growth, nitrogen absorption and metabolism, we generated *AMT1-3*-overexpressing plants *via* the *Agrobacterium-*mediated transformation method using the *CaMV*35S promoter in this study, and analyzed the growth phenotype, yield, leaf SPAD (Soil and Plant Analyzer Development) value, photosynthesis, carbon and nitrogen metabolic status and gene expression profiles under four different N levels (0× N, 0.1× N, 1× N and 5× N) at both tillering and heading stages. Results revealed that the overexpressing of *AMT1-3* gene altered rice growth and development, yield, C/N ratio, soluble proteins and carbohydrates, carbon and nitrogen metabolites, gene expression patterns in transgenic plants when compared to the wild-type plants. Moreover, *AMT1-3* may act as a signal sensor to regulate plant growth in addition to its function in NH_4_^+^ uptake in the root. Our results provided a new understanding of the function of *AMT1-3* genes in rice growth, yield, carbon and nitrogen metabolism, and a reference for the molecular breeding of the high nitrogen use efficiency rice cultivar using transgenic strategy.

## 2. Results

### 2.1. Accumulated AMT1-3 mRNA Transcripts in T_0_ Transgenic Plants

**Figure 1:**
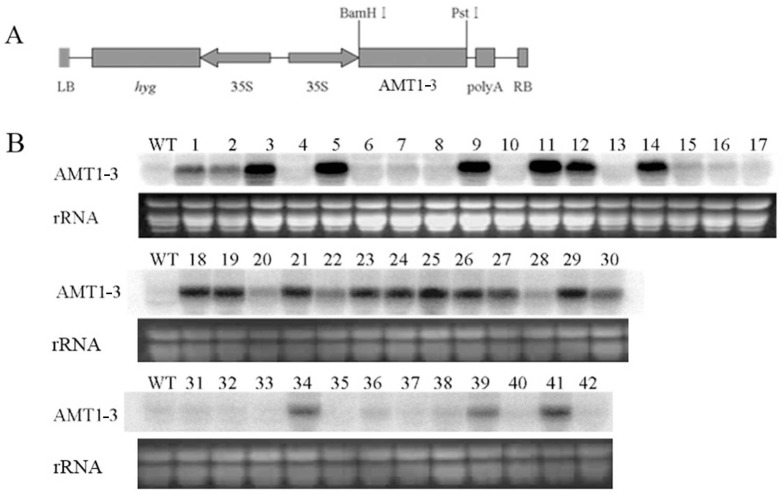
Generation of transgenic plants that overexpressing the *AMT1-3* gene. (**A**) The construct of the plasmid containing a derivative of the *CaMV* 35S promoter (35S), *AMT1-3* and the poly A terminator between the right (RB) and the left (LB) borders of the T-DNA. The *hygromycin resistance* gene (*hyg*) was located between the LB and the 35S promoter; (**B**) Northern blot analysis of the *AMT1-3* mRNA transcriptional levels in 42 positive transgenic plants of the T_0_ generation and the wild-type plants (WT).

**Figure 2:**
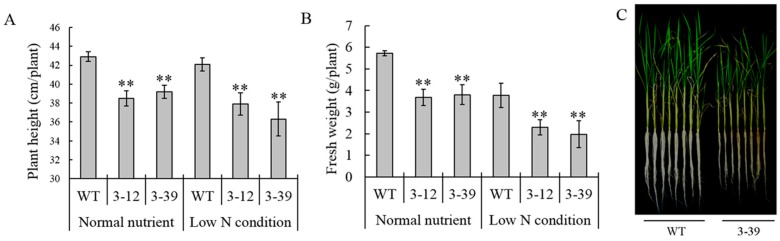
Phenotype analysis at the seedling stage. The growth phenotype (**A**), plant height (**B**) and plant fresh weight (**C**) of the *AMT1-3*-overexpressing plants (3–12, 3–39) and wild type plants (WT) at the seedling stage. Values are the mean ± s.d. of eight randomly selected plants. ** indicates the significant difference at the level of *p* = 0.01.

The complete mRNA sequence of *OsAMT1-3* (AF289479) was identified in the NCBI GenBank database using a key word search. The *OsAMT1-3* gene was amplified from the rice (Zhonghua 11, *Japonica*) genome and ligated into the pCAMBIA 1301S vector, which contained a *hygromycin resistance* gene driven by the *CaMV*35S promoter. The construct was then transformed into Zhonghua 11 using the *Agrobacterium*-mediated transformation method (Figure 1A). More than 50 independent T_0_ transformants were generated, and 42 positive transformants were detected by PCR-directed analysis of the *hygromycin resistance* gene. Northern blot analysis of the transgene in these independent positive transgenic plants revealed that 21 (50%) transgenic plants harbored higher *OsAMT1-3* mRNA levels than the wild-type Zhonghua 11 (Figure 1B). Southern blot analysis showed 1–2 copies of transgene existed in the *OsAMT1-3*-overexpressing plants (Figures S1). To examine the effect of higher *OsAMT1-3* expression on plant growth, T_1_ progeny of the 21 *OsAMT1-3*-overexpressing plants and wild-type plants were grown hydroponically in normal and low nitrogen (0.1× N) nutrient solutions. Six-week-old plants were harvested to measure the plant height and fresh weight. Generally, transgenic lines exhibited a poorer growth than the wild-type plants under both normal and low nitrogen conditions. Figure 2 showed that the plant height and fresh weight of two transgenic lines (3–12, 3–39) were significantly (*p* < 0.01) decreased when compared with wild-type plants under both normal and low nitrogen conditions. To avoid confounding effects of the excessively higher *OsAMT1-3* gene expression on plant growth, we selected the T_2_ generation of transgenic line 3–39 with single copy of transformed *AMT1-3*, which displayed higher *OsAMT1-3* levels than wild-type plants but were relatively lower among the 21 transgenic plants, for further analysis and was named *AMT1-3*-overexpressing plants.

### 2.2. Effect of AMT1-3-Overexpressing on Growth Phenotype and Yield

**Figure 3:**
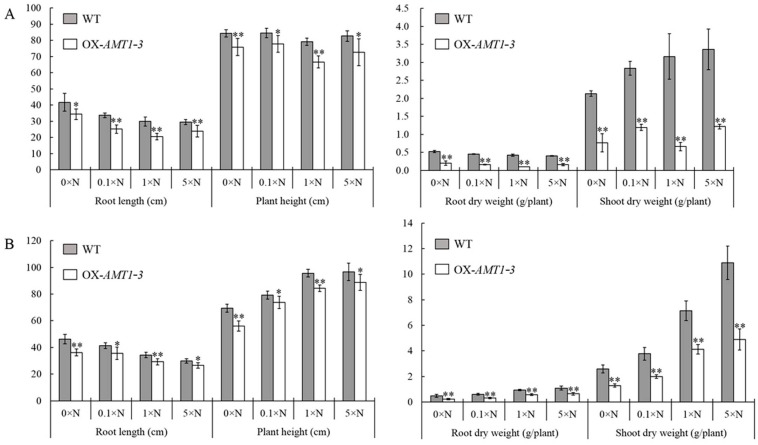
Phenotype analysis at the tillering and heading stages. The root length, plant height, root and shoot dry weight in the *AMT1-3*-overexpressing plants (we renamed 3–39 as OX-*AMT1-3*) and wild type plants (WT) at the tillering stage (**A**) and the heading stage (**B**) under 0× N, 0.1× N, 1× N and 5× N conditions. Values are the mean ± s.d. of ten randomly selected plants. *, ** indicate the significant differences at the level of *p* = 0.05 and *p* = 0.01, respectively.

As the *AMT1-3*-overexpressing plants displayed a poor growth phenotype at the seedling stage mentioned above, we analyzed the root length, plant height, root and shoot dry weight, leaf SPAD value and photosynthetic parameters of *AMT1-3*-overexpressing plants and wild-type plants at both tillering and heading stages grown hydroponically under four different nitrogen levels (0× N, 0.1× N, 1× N and 5× N) to test the poor growth phenotype at both vegetative and productive stages in more detail. The *AMT1-3*-overexpressing and wild-type plant yields at a mature growth stage in pots were also tested. The results revealed a significant (*p* < 0.05) decline in the root length and plant height and in the root and shoot dry weight in the *AMT1-3*-overexpressing plants compared to wild-type plants at both tillering and heading stages under the 0× N, 0.1× N, 1× N and 5× N conditions (Figure 3). At the tillering stage, a 17.7%–31.2%, 8%–15.8%, 60.7%–77.3% and 58%–79.1% decrease in the root length, plant height and the root and shoot dry weights of *AMT1-3*-overexpressing plants were observed, respectively (Figure 3A). At the heading stage, a 11.3%–21.6%, 6.9%–19.2%, 38.6%–55.2% and 42.3%–55.1% decrease in the root length, plant height and the root and shoot dry weights of *AMT1-3*-overexpressing plants were observed, respectively (Figure 3B). The leaf SPAD value and photosynthetic parameters did not significantly differ between the *AMT1-3*-overexpressing plants and wild-type plants, except for significant (*p* < 0.05) decreases in the photosynthetic rate and stomatal conductance in the leaves of *AMT1-3*-overexpressing plants compared with wild-type plants at the heading stage under the 1× N condition (Table 1). A yield analysis at the mature stage revealed decreases of 45.6%, 8.5%, 9.5% and 26.4% in *AMT1-3*-overexpressing plants compared with wild-type plants under the 0× N, 0.1× N, 1× N and 5× N conditions, respectively (Table 2). These results indicated that *AMT1-3* gene overexpressing could severely affect plant growth and development at both vegetative and productive stages under different nitrogen levels.

**Table 1:** The leaf SPAD value and photosynthetic parameters in the *AMT1-3*-overexpressing plants (OX-*AMT1-3*) and wild type plants (WT) at the tillering stage and the heading stage under 0× N, 0.1× N, 1× N, 5× N conditions.

Treatment	SPAD		Photosynthesis Parameters at Heading Stage			
	Tillering Stage	Heading Stage	Photosynthetic Rate (μmol CO_2_·m^−2^·s^−2^)	Stomatal Conductance (mmol·m^−2^·s^−1^)	Intercellular CO_2_ Concentration (μL·L^−1^)	Transpiration Rate (mmol H_2_O·m^−2^·S^−1^)
**0× N**						
WT	34.3 ± 2.4	32.7 ± 1.7	13.24 ± 1.47	0.28 ± 0.04	280.48 ± 1.12	7.54 ± 0.65
OX-*AMT1-3*	34.3 ± 2.5	33.0 ± 1.4	11.84 ± 2.34	0.25 ± 0.05	280.16 ± 8.55	6.71 ± 0.95
**0.1× N**						
WT	40.7 ± 2.0	40.7 ± 2.2	10.58 ± 1.64	0.29 ± 0.02	297.52 ± 10.16	7.24 ± 0.25
OX-*AMT1-3*	42.3 ± 3.4	40.4 ± 1.2	12.88 ± 1.59	0.30 ± 0.05	287.51 ± 11.30	7.20 ± 0.71
**1× N**						
WT	43.6 ± 2.0	47.1 ± 0.5	16.07 ± 1.78	0.41 ± 0.04	290.97 ± 8.26	7.49 ± 0.47
OX-*AMT1-3*	44.4 ± 1.3	46.5 ± 1.3	12.49 ± 1.76 *	0.27 ± 0.09 **	278.65 ± 23.78	6.09 ± 1.47
**5× N**						
WT	44.4 ± 1.5	48.4 ± 1.3	19.12 ± 3.45	0.53 ± 0.05	305.62 ± 15.74	7.85 ± 0.17
OX-*AMT1-3*	43.6 ± 4.0	47.4 ± 1.3	16.95 ± 2.39	0.48 ± 0.09	300.49 ± 14.01	7.86 ± 1.04

Values are mean ± s.d. from ten randomly selected plants. *, ** indicate the significant differences at the level of *p* = 0.05 and *p* = 0.01, respectively.

**Table 2:** The yield and its components in the *AMT1-3*-overexpressing plants (OX-*AMT1-3*) and wild type plants (WT) under 0× N, 0.1× N, 1× N, 5× N conditions.

Treatment	Panicals/Plant	Filled Grains/Panicle	Seed Rate (%)	Thousand Grains Weight (g)	Yield (g/Plant)
**0× N**					
WT	3.4 ± 0.3	19.1 ± 1.5	35.1 ± 1.5	24.46 ± 0.65	1.60 ± 0.11
OX-*AMT1-3*	2.5 ± 0.4 *	15.6 ± 2.6	33.7 ± 1.2	22.89 ± 0.94	0.87 ± 0.05 **
**0.1× N**					
WT	4.0 ± 0.2	21.4 ± 3.5	36.4 ± 4.3	23.05 ± 0.74	2.00 ± 0.44
OX-*AMT1-3*	3.5 ± 0.2	21.9 ± 3.8	33.6 ± 4.8	23.68 ± 0.91	1.83 ± 0.23
**1× N**					
WT	11.5 ± 1.1	44.9 ± 4.9	66.9 ± 5.9	24.98 ± 0.21	12.78 ± 0.42
OX-*AMT1-3*	11.6 ± 0.6	39.5 ± 4.3	64.8 ± 6.1	25.35 ± 0.31	11.56 ± 0.87
**5× N**					
WT	14.6 ± 0.9	32.5 ± 2.3	65.9 ± 10.9	22.77 ± 0.75	10.80 ± 0.41
OX-*AMT1-3*	15.6 ± 0.7	23.3 ± 0.6 **	54.0 ± 4.4	21.87 ± 0.91	7.95 ± 0.26 **

Values are mean ± s.d. from ten randomly selected plants. *, ** indicate the significant differences at the level of *p* = 0.05 and *p* = 0.01, respectively.

### 2.3. Effect of AMT1-3-Overexpressing on Nitrogen Uptake and Accumulation

**Figure 4:**
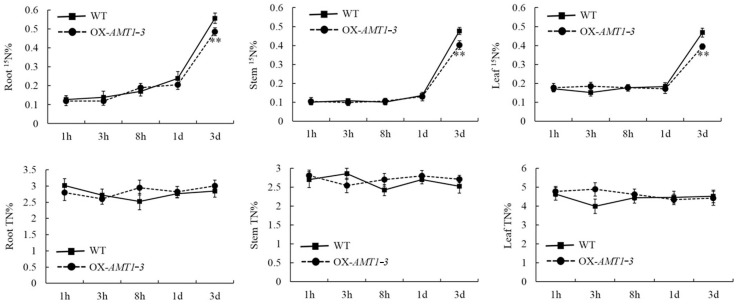
Nitrogen uptake analysis. The ^15^N (^15^N%) and total nitrogen content (TN%) in the roots, stems and leaves of the *AMT1-3*-overexpressing plants (we renamed 3–39 as OX-*AMT1-3*) and wild type plants (WT) at 1, 3, 8 h, 1 and 3 day after NH_4_Cl in the nutrient solution was replaced with ^15^NH_4_Cl during the tillering stage. Values are the mean ± s.d. of six randomly selected plants. ** indicates the significant difference at the level of *p* = 0.01.

As *AMT1-3* is one of the high affinity ammonium transporter genes involved in NH_4_^+^ uptake in rice roots, we analyzed the ^15^N and total N contents in roots, stems and leaves of *AMT1-3*-overexpressing plants and wild-type plants. We employed the ^15^N tracer assay at the tillering stage to assess for differences in the nitrogen uptake ability in the root and in the nitrogen transport ability from the root to the stem and from the stem to the leaf between *AMT1-3*-overexpressing plants and wild-type plants. The results indicated that *AMT1-3*-overexpressing plants harbored lower ^15^N contents in the roots, stems and leaves, especially three days after NH_4_Cl in the nutrient solution was refreshed by ^15^NH_4_Cl (Figure 4). Furthermore, we analyzed the total carbon and nitrogen contents in the roots, stems and leaves of *AMT1-3*-overexpressing plants and wild-type plants under the 0× N, 0.1× N, 1× N and 5× N conditions at this stage. Because we performed two independent assays, the plant materials differed from the materials used in the ^15^N tracer assay. The results indicated significant (*p* < 0.05) decreases in the leaf carbon and nitrogen contents in *AMT1-3*-overexpressing plants compared with wild-type plants under the 0× N, 0.1× N, 1× N and 5× N conditions (Table 3). There were 4.6%, 9.9%, 5.9% and 5.8% decreases in the leaf carbon content, whereas decreases of 4.7%, 13.4%, 15.5% and 8.6% in the leaf nitrogen content under the 0× N, 0.1× N, 1× N and 5× N conditions were observed, respectively, which resulted in leaf carbon/nitrogen ratio increases of 11.7%, 11.6% and 9.4% under the 0.1× N, 1× N and 5× N conditions, respectively (Table 3). These results suggest that *AMT1-3*-overexpressing plants exhibited a poor nitrogen transport ability. The nitrogen transport from stems to leaves was especially attenuated, which caused a higher carbon/nitrogen ratio in the leaves.

**Table 3:** The carbon content (C%), nitrogen content (N%) and carbon/nitrogen ratio (C/N) in the roots, stems and leaves of the *AMT1-3*-overexpressing plants (OX-*AMT1-3*) and wild type plants (WT) at the tillering stage under 0× N, 0.1× N, 1× N, 5× N conditions.

Treatment		C%			N%			C/N	
	Root	Stem	Leaf	Root	Stem	Leaf	Root	Stem	Leaf
**0× N**									
WT	38.54 ± 0.60	38.26 ± 0.43	40.10 ± 0.20	1.76 ± 0.06	0.96 ± 0.04	2.14 ± 0.03	21.85 ± 0.77	39.79 ± 1.05	18.71 ± 0.25
OX-*AMT1-3*	36.36 ± ND	36.07 ± 1.63	38.25 ± 0.39 **	1.62 ± ND	1.02 ± 0.03	2.04 ± 0.09	22.39 ± ND	35.26 ± 0.61 **	18.80 ± 0.67
**0.1× N**									
WT	38.47 ± 0.07	38.88 ± 0.10	40.52 ± 0.16	2.10 ± 0.14	1.40 ± 0.11	2.91 ± 0.18	18.34 ± 1.17	27.80 ± 2.20	13.94 ± 0.81
OX-*AMT1-3*	39.13 ± 0.76	37.53 ± 0.39	36.51 ± 0.35 **	2.13 ± ND	1.39 ± 0.07	2.52 ± 0.15 *	17.17 ± ND	26.97 ± 1.59	15.57 ± 0.85 *
**1× N**									
WT	37.48 ± 0.60	37.00 ± 0.36	41.46 ± 0.25	2.72 ± 0.004	2.44 ± 0.08	3.75 ± 0.08	13.77 ± 0.20	15.19 ± 0.49	11.05 ± 0.16
OX-*AMT1-3*	ND	36.21 ± 0.46	39.01 ± 0.13 **	ND	2.37 ± 0.21	3.17 ± 0.14 **	ND	15.36 ± 1.24	12.33 ± 0.59 *
**5× N**									
WT	37.69 ± 0.61	36.96 ± 0.18	41.46 ± 0.25	2.85 ± 0.16	3.04 ± 0.08	3.98 ± 0.08	13.27 ± 1.00	12.15 ± 0.31	10.41 ± 0.15
OX-*AMT1-3*	37.21 ± ND	36.08 ± 0.78	39.04 ± 0.72 **	2.56 ± ND	2.96 ± 0.02	3.43 ± 0.23 *	14.52 ± ND	12.18 ± 0.21	11.39 ± 0.60 *

Values are mean ± s.d. from three biological replications. *, ** indicate the significant differences at the level of *p* = 0.05 and *p* = 0.01, respectively. ND: no data.

### 2.4. Effect of AMT1-3-Overexpressing on Soluble Proteins and Carbohydrates Concentrations

**Figure 5:**
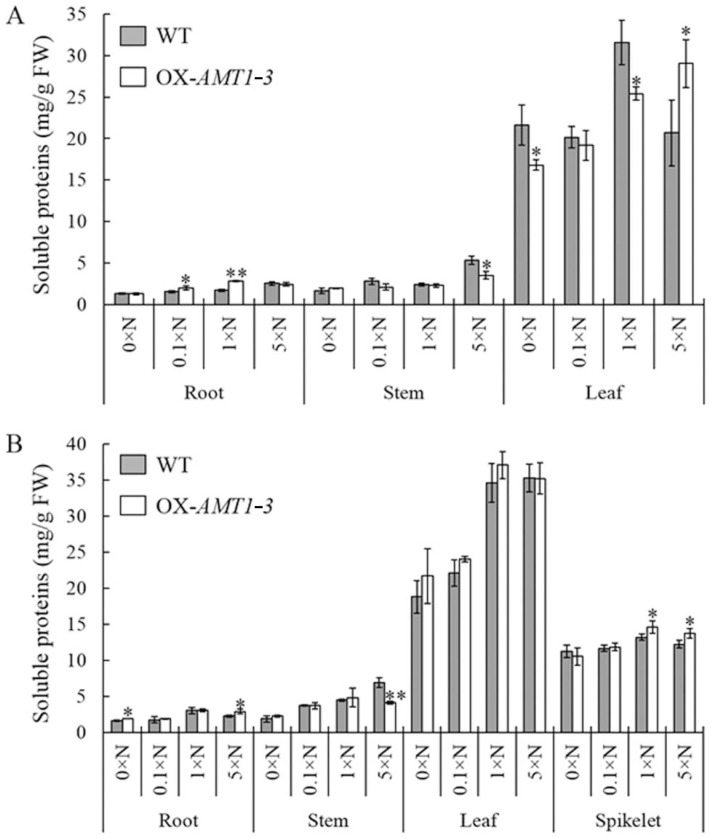
Soluble proteins analysis. The concentration of soluble proteins in the roots, stems and leaves of the *AMT1-3*-overexpressing plants (we renamed 3-39 as OX-*AMT1-3*) and wild type plants (WT) at the tillering stage (**A**) and the heading stage (**B**) under 0× N, 0.1× N, 1× N and 5× N conditions. Values are the mean ± s.d. from three biological replications. *, ** indicate the significant differences at the level of *p* = 0.05 and *p* = 0.01, respectively.

**Figure 6:**
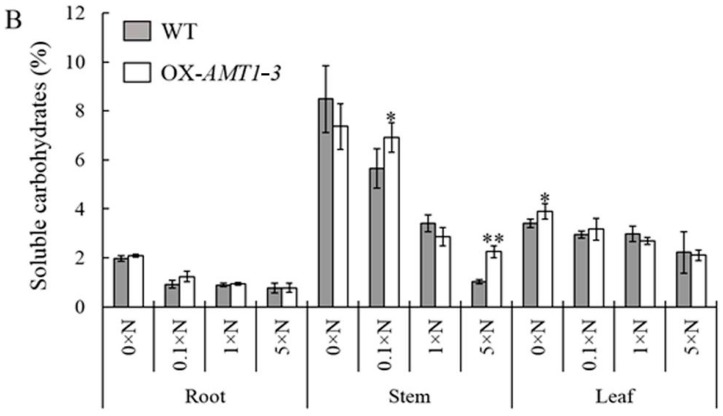

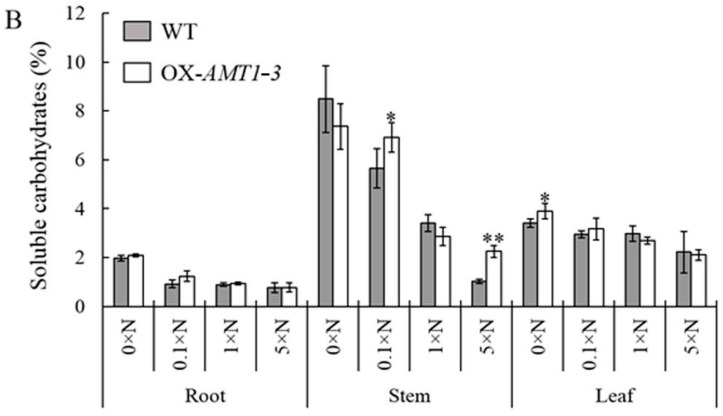
Soluble carbohydrates analysis. The concentration of soluble carbohydrates in the roots, stems and leaves of the *AMT1-3*-overexpressing plants (we renamed 3–39 as OX-*AMT1-3*) and wild type plants (WT) at the tillering stage (**A**) and the heading stage (**B**) under 0× N, 0.1× N, 1× N and 5× N conditions. Values are the mean ± s.d. from three biological replications. *, ** indicate the significant differences at the level of *p* = 0.05 and *p* = 0.01, respectively.

As the nitrogen and carbon contents were changed by the overexpressing of *AMT1-3* mentioned above, we wanted to evaluate the differences of carbon and nitrogen metabolic status between *AMT1-3*-overexpressing plants and wild-type plants. Therefore, we assessed the concentrations of soluble proteins and carbohydrates in the roots, stems and leaves of *AMT1-3*-overexpressing plants and wild-type plants at both tillering and heading stages under the 0× N, 0.1× N, 1× N and 5× N conditions. Our results demonstrated that most of the soluble proteins were in the leaves, whereas most of the soluble carbohydrates resided in the stems. Interestingly, the soluble proteins increased with increasing nitrogen levels, whereas an opposite trend was observed for soluble carbohydrates, which decreased with increasing nitrogen levels at both the tillering and heading stages (Figure 5 and Figure 6). The soluble protein and carbohydrate concentrations in *AMT1-3*-overexpressing plants were noticeably distinct from those of wild-type plants (Figure 5 and Figure 6). Compared to wild type plants, there were 18.5%, 4.9%, 14.3% decreases and 22.3% increase of soluble proteins in *AMT1-3*-overexpressing plants at tillering stage under 0× N, 0.1× N, 1× N and 5× N conditions, respectively. While an opposite change pattern displayed at heading stage that there were 8.6%, 5.8%, 7.8% increases and 1.3% decrease of soluble proteins in *AMT1-3*-overexpressing plants under 0× N, 0.1× N, 1× N and 5× N conditions, respectively (data not show here). For soluble carbohydrates, 15.5%, 18.5%, 2.1% and 10.4% decreases displayed in *AMT1-3*-overexpressing plants at tillering stage, while 3.8% decrease, 19.0% increase, 10.6% decrease and 28.1% increase displayed in *AMT1-3*-overexpressing plants at heading stage under 0× N, 0.1× N, 1× N and 5× N conditions, respectively (data not show here). Additionally, there was a certain change in the concentrations of root, stem and leaf soluble proteins and carbohydrates in *AMT1-3*-overexpressing plants when compared to wild type plants (Figure 5 and Figure 6). For example, soluble proteins analysis at the tillering stage revealed significant (*p* < 0.05) increases in the root under the 0.1× N (30.6%) and 1× N (66.9%) conditions and in the leaf under the 5× N (40.4%) condition, as well as significant (*p* < 0.05) decreases in the stem under the 5× N (34.3%) condition and in the leaf under the 0× N (22.2%) and 1× N (19.5%) conditions. However, an analogous analysis at the heading stage revealed significant (*p* < 0.05) increases in the root under the 0× N (14.1%) and 5× N (29.8%) conditions and in the spikelet under the 1× N (10.5%) and 5× N (12.4%) conditions, as well as significant (*p* < 0.05) decreases in the stem under the 5× N (40.8%) condition (Figure 5). An analysis of soluble carbohydrates at the tillering stage demonstrated significant (*p* < 0.05) increases in the root under the 5× N (14.5%) condition and in the stem under the 1× N (25.9%) condition, as well as significant (*p* < 0.05) decreases in the stem under the 5× N (34.3%) condition and in the leaf under the 0× N (29.3%) and 1× N (34.0%) conditions. An analogous analysis at the heading stage indicated significant (*p* < 0.05) increases in the stem under the 0.1× N (22.6%) and 5× N (119.3%) conditions and in the leaf under the 0× N (14.1%) condition (Figure 6). These results suggest that the overexpressing of the *AMT1-3* gene altered carbon and nitrogen metabolic statuses in transgenic plants.

### 2.5. Effect of AMT1-3-Overexpressing on Carbon and Nitrogen Metabolites

**Figure 7:**
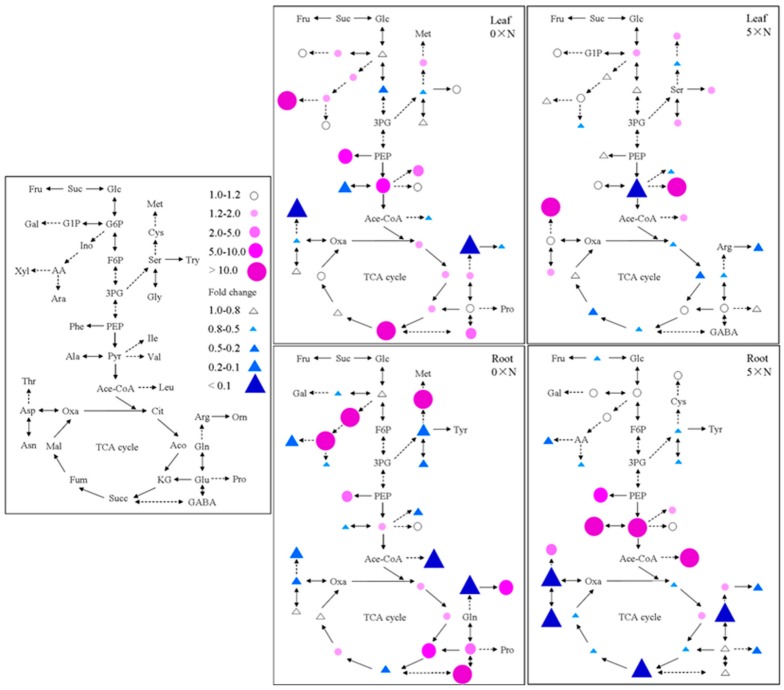
Metabolites analysis. Fold change corresponds to the ratio of the concentration of individual metabolites involved in carbon and nitrogen metabolism in the *AMT1-3*-overexpressing plants relative to the wild type plants for the leaves and roots at the tillering stage under 0× N and 5× N conditions. Glc, glucose; Suc, sucrose; Fru, Fructose; F6P, Frutose-6-P; G6P, Glucose-6-P; G1P, Glucose-1-P; Gal, galactose; Ino, Inositol; AA, Ascorbic acid; Ara, Arabinose; Xyl, Xylitol; 3PG, 3-P-glycerate; PEP, P-enolpyruvate; Pyr, Pyruvate; Ace-CoA, acetyl-CoA; Cit, Citrate; Aco, Aconitase; KG, Ketoglutarate; Succ, Succinate; Fum, Fumarate; Mal, Malate; Oxa, oxaloacetate; Glu, Glutamate; Gln, Glutamine; Arg, Arginine; Pro, Proline; Orn, Ornithine; GABA, Aminobutyric; Asp, Aspartate; Asn, Asparagine; Ile, Isoleucine; Met, Methionine; Thr, Threonine; Ala, Alanine; Val, Valine; Leu, Leucine; Phe, Phenylalanine; Try, Tryptophan; Ser, Serine; Gly, Glycine; Cys, Cysteine. Red dots indicate increased metabolites and blue triangles indicate decreased metabolites.

To study the individual metabolites involved in the carbon and nitrogen metabolic pathways, we analyzed the sugars, organic acids and free amino acids in the roots and leaves of *AMT1-3*-overexpressing plants and wild-type plants at the tillering stage under the 0× N and 5× N conditions. Figure 7 and supplementary Figure S2 displays the fold change that corresponds to the ratio of *AMT1-3*-overexpressing plants/wild-type plants calculated by the concentration of these individual metabolites. For the total sugars, total organic acids and total free amino acids, the results indicated similar patterns between the roots and leaves, where the total sugars and total free amino acids decreased and the total organic acids increased in *AMT1-3*-overexpressing plants under the 0× N condition, and the total sugars and total organic acids decreased and the total free amino acids increased in *AMT1-3*-overexpressing plants under the 5× N condition (data not shown). The fold change of individual sugars, organic acids and free amino acids in *AMT1-3*-overexpressing plants compared with wild-type plants indicates that *AMT1-3* overexpressing yielded larger metabolite content variations in the roots than the leaves and under the 0× N condition than the 5× N condition. In *AMT1-3*-overexpressing plant leaves, when compared with wild-type plants, dramatic increases in xylitol (>21.9-fold) and succinate (>277.6-fold) and dramatic decreases in benzoic acid (<0.01-fold), arginine (<0.04-fold) and threonine (<0.03-fold) were observed under the 0×N condition. However, dramatic increases in threonine (>42.8-fold) and valine (>32.5-fold) and dramatic decrease in pyruvate (<0.07-fold) were observed under the 5× N condition (Figure 7 and Figure S2). In *AMT1-3*-overexpressing plant roots, when compared with wild-type plants, dramatic increases in inositol (>21.2-fold), ascorbic acid (>96.5-fold), aminobutyric acid (>526.6-fold) and cysteine (>20.0-fold) and dramatic decreases in arginine (<0.07-fold) and leucine (<0.001-fold) were observed under the 0× N condition. However, dramatic increases in benzoic acid (>14.0-fold), pyruvate (>684.7-fold), alanine (>63.3-fold) and leucine (>21.0-fold) and dramatic decreases in succinate (<0.10-fold), glutamine (<0.06-fold), aspartate (<0.05-fold) and asparagine (<0.005-fold) were observed under the 5× N condition (Figure 7 and Figure S2).

### 2.6. Effect of AMT1-3-Overexpressing on Gene Expression Level

**Figure 8:**
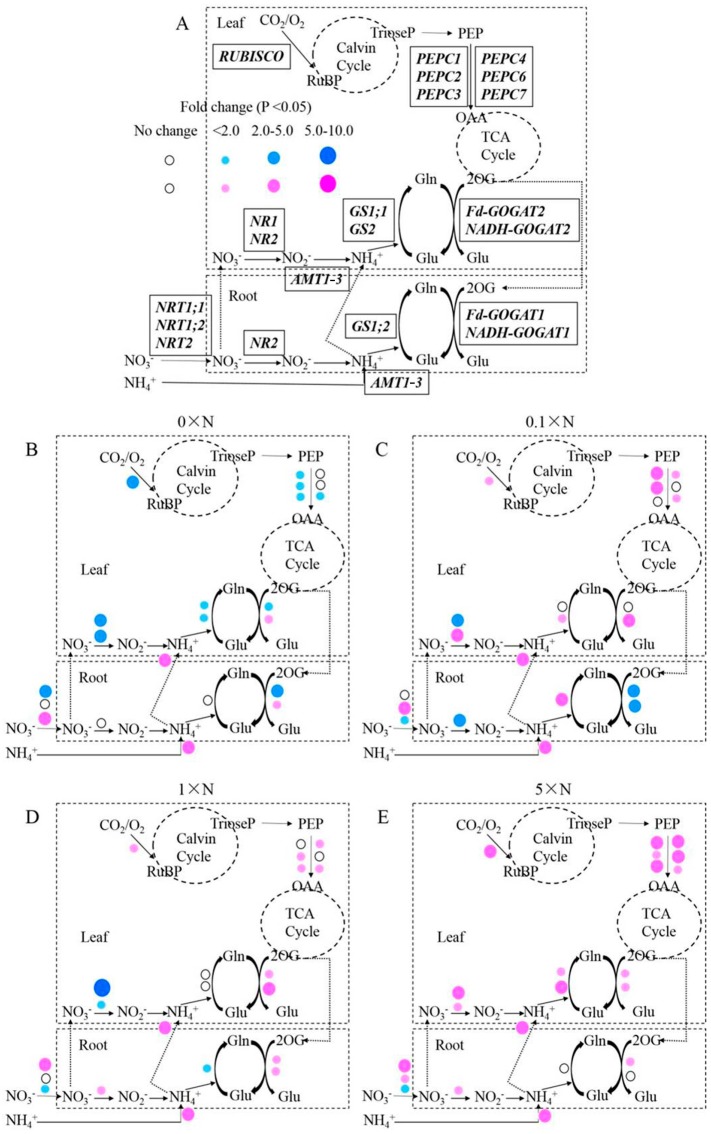
Gene expression analysis. Fold change corresponds to the ratio of the gene expression level in the *AMT1-3*-overexpressing plants relative to the wild type plants. (**A**) Diagrammatic representation of the key genes involved in the carbon and nitrogen metabolic pathway in rice plants. NRT, nitrate transporter; NR, nitrate reductase; GS, glutamine synthetase; GOGAT, glutamate synthase; RUBISCO, ribulose-1,5-bisphosphate carboxylase/oxygenase; PEPC, phosphoenolpyruvate carboxylase. Prominent changes in the gene expression level in the *AMT1-3*-overexpressing plants compared to wild type plants at the tillering stage under 0× N (**B**); 0.1× N (**C**); 1× N (**D**) and 5× N (**E**) conditions. Red and blue dots indicate up- and down-regulated genes, respectively.

To analyze the impact of higher *AMT1-3* mRNA levels on the expression patterns of key genes involved in carbon and nitrogen metabolism, the expression levels of genes encoding NRT (nitrate transporter), NR (nitrate reductase), GS (glutamine synthetase), GOGAT (glutamate synthase), RUBISCO (ribulose-1,5-bisphosphate carboxylase/oxygenase) and PEPC (phosphoenolpyruvate carboxylase) were analyzed by q-RT PCR. Figure 8A displays these genes in the carbon and nitrogen metabolic pathways in rice plants. Supplementary Table S1 lists the fold changes that correspond to the ratio of *AMT1-3*-overexpressing plants/wild-type plants calculated by the relative gene expression level in roots and leaves at the tillering stage under the 0× N, 0.1× N, 1× N and 5× N conditions. The results demonstrated that although *AMT1-3* was constitutively overexpressing under the 35S promoter, these genes displayed distinct expression patterns under different nitrogen levels.

Under the 0× N condition, compared with wild-type plants, the roots of *AMT1-3*-overexpressing plants displayed significantly decreased *NTR1*;*1* and *Fd-GOGAT1* levels (*p* < 0.01) and significantly increased *NRT2* and *NADH-GOGAT1* levels (*p* < 0.01), whereas the leaves of *AMT1-3*-overexpressing plants displayed significantly decreased levels for most of the genes assayed (*NR1*, *NR2*, *GS1;1*, *GS2*, *Fd-GOGAT2*, *RUBISCO*, *PEPC1*, *PEPC2*, *PEPC3*, *PEPC7*) (*p* < 0.05), except one gene (*NADH-GOGAT2*), which significantly increased (*p* < 0.01) (Figure 8B, Supplementary Table S1). Under the 0.1× N condition, compared with wild-type plants, the roots of *AMT1-3*-overexpressing plants displayed significantly increased *NRT1;2* and *GS1;2* levels (*p* < 0.01) and significantly decreased *NRT2*, *NR2*, *Fd-GOGAT1* and *NADH-GOGAT1* levels (*p* < 0.05), which differed from the results obtained under the 0× N condition, where the leaves of *AMT1-3*-overexpressing plants displayed significant increases in most of the genes assayed (*NR2*, *GS2*, *NADH-GOGAT2*, *RUBISCO*, *PEPC1*, *PEPC2*, *PEPC4*, *PEPC7*) (*p* < 0.05), except one gene (*NR1*), which significantly decreased (*p* < 0.01) (Figure 8C, Supplementary Table S1). Under the 1× N condition, compared with wild-type plants, the roots of *AMT1-3*-overexpressing plants displayed significantly increased *NRT1;1*, *NR2*, *Fd-GOGAT1* and *NADH-GOGAT1* levels (*p* < 0.05) and significantly decreased *NRT2* and *GS1;2* levels (*p* < 0.05), whereas the leaves of *AMT1-3*-overexpressing plants displayed similar results to those obtained under the 0.1× N condition, where most of the genes significantly increased (*Fd-GOGAT2*, *NADH-GOGAT2*, *RUBISCO*, *PEPC2*, *PEPC3*, *PEPC4*, *PEPC7*) (*p* < 0.05) , except the *NR* genes (*NR1* and *NR2*), which significantly decreased (*p* < 0.05) (Figure 8D, Supplementary Table S1). Under the 5× N condition, the expression levels of the detected genes were significantly increased (*p* < 0.05) in the roots and leaves of *AMT1-3*-overexpressing plants compared with wild-type plants, except one gene (*NRT2*), which significantly decreased (*p* < 0.05) (Figure 8E, Table S1).

## 3. Discussion

As *OsAMT1* members, including *OsAMT1-1*, *OsAMT1-2* and *OsAMT1-3*, have been characterized as high-affinity NH_4_^+^ transport genes in rice [13,35], we expected to obtain *OsAMT1*-overexpressing transgenic rice plants with higher ammonium uptake capacity, improved nitrogen use efficiency and a low nitrogen tolerance using the *Agrobacterium-*mediated transformation method. Although we generated *AMT1-2*-, *AMT1-3*-overexpressing plants, we failed to obtain *AMT1-1*-overexpressing plants. Unexpectedly, *AMT1-3*-overexpressing plants displayed poor growth, but no significant differences between *AMT1-2*-overexpressing plants and wild-type plants were observed. In order to understand why poor growth phenotype exhibited in *AMT1-3*-overexpressing transgenic rice plants and evaluate the effect of *AMT1-3* gene on the rice growth, nitrogen absorption and metabolism, we analyzed the growth phenotype, yield, carbon and nitrogen metabolic status and gene expression profiles of the *OsAMT1-3*-overexpressing rice and wild-type Zhonghua 11 under four different N levels (0× N, 0.1× N, 1× N and 5× N) at both tillering and heading stages in detail. Results showed that despite the higher *AMT1-3* mRNA levels in transgenic plants, the root length, plant height, root and shoot biomass and yield were significantly decreased in *AMT1-3*-overexpressing plants compared with wild-type plants (Figure 2 and Figure 3; Table 2). The contents of total carbon and nitrogen, soluble proteins and carbohydrates, individual metabolites (such as sugars, organic acids, amino acids), the expression patterns of key genes involved in carbon and nitrogen metabolic pathway changed significantly in *AMT1-3*-overexpressing plants (Figure 5, Figure 6, Figure 7 and Figure 8; Table 3). The discordant of carbon and nitrogen metabolic status in transgenic plants caused by the *OsAMT1-3* overexpressing may be the main reason for poor growth and yield in our investigation. Similar results were reported that overexpressing of *OsAMT1-1* gene caused decreased shoot and root biomass in transgenic lines during seedling and early vegetative stage, especially when grown under high ammonium nutrition [53,54]. However, completely different results were reported by Ranathunge *et al*. [55] that transgenic rice lines overexpressing the *OsAMT1-1* gene had higher plant growth rate and grain yield, especially under suboptimal NH_4_^+^ levels.

As we known, both carbon and nitrogen nutrients are essential for various cellular functions. Recently, cellular carbon and nitrogen metabolism was demonstrated to be tightly coordinated [8,9,10,11,56]. In addition to independent utilization, the coordination and optimal functioning of the metabolic pathways governing nitrogen and carbon assimilation in plants were demonstrated to be critical for plant growth and, ultimately, biomass accumulation and yield production [10,56]. Maintaining an appropriate balance or carbohydrate to nitrogen metabolite ratio in the cell, which is referred to as the “carbon/nitrogen balance”, is extremely important for regulating plant growth, development and production yield [8,9,11]. In our study, we found 4.6%–9.9% and 4.7%–15.5% decreases in *AMT1-3*-overexpressing plant leaf C and N content, respectively, which resulted in a higher (9.4%–11.7% increases) leaf C/N ratio in *AMT1-3*-overexpressing plant leaves (Table 3). Additionally, distinct changes between the soluble protein and carbohydrate concentrations were observed in *AMT1-3*-overexpressing plants (Figure 5 and Figure 6). Therefore, combined with our results, we hypothesized that the carbon and nitrogen metabolic imbalance caused by *AMT1-3* overexpressing attributed to the poor growth and yield of transgenic plants.

To further study the impact of higher *AMT1-3* mRNA levels at both the physiological and molecular level, we analyzed the individual carbon and nitrogen metabolites and the gene expression levels in the roots and leaves of *AMT1-3*-overexpressing plants and wild-type plants. Consistent results of the metabolite concentrations and gene expression levels were showed in this study. Under the 0× N growth condition, given an almost complete lack of nitrogen (a residual amount of nitrogen may reside in water) in the nutrient solution, the nitrogen uptake, reduction and assimilation levels declined in leaves. The expression level of *NRT1;1*, which encodes a low-affinity NO_3_^−^ transporter [57], was repressed, whereas *NRT2*, which encodes a high-affinity NO_3_^−^ transporter [58], was induced in the root. Although the *AMT1-3* gene was overexpressing under the 35S promoter, the NH_4_^+^ concentration in roots and leaves significantly decreased. Together with the lower NO_3_^−^ uptake and transport by NRTs in roots and leaves, the expression levels of the genes (*NR1*, *NR2*, *GS1;1*, *GS2*, *Fd-GOGAT1* and *Fd-GOGAT2*) involved in NO_3_^−^ reduction and NH_4_^+^ assimilation were significantly decreased, which resulted in reduced total free amino acids and soluble proteins (Figure 5, Figure 7 and Figure 8). During NH_4_^+^ assimilation, numerous organic acids provided carbon skeletons to produce a variety of amino acids [59]; therefore, an accumulation of total organic acids may have been observed because of the decreased nitrogen assimilation status in leaves of *AMT1-3*-overexpressing plants (Figure 8). The expression levels of *RUBISCO*, *PEPC1*, *PEPC2*, *PEPC3* and *PEPC7* were decreased, which lead to a decline in total sugars and soluble carbohydrates in leaves of *AMT1-3*-overexpressing plants (Figure 6, Figure 7 and Figure 8). Under the 5× N growth condition, given the much higher nitrogen content in the nutrient solution, higher levels of nitrogen uptake, reduction and assimilation were observed in both roots and leaves of *AMT1-3*-overexpressing plants. The expression of low-affinity NO_3_^−^ transporter genes (*NRT1;1* and *NRT1;2*) [57] were induced, whereas the expression of the high-affinity NO_3_^−^ transporter gene *NRT2* [58] was repressed in the root, which resulted in more NO_3_^−^ absorption and higher expression of genes (*NR1*, *NR2*, *GS1;1*, *GS2*, *Fd-GOGAT1*, *Fd-GOGAT2* and *NADH-GOGAT2*) involved in NO_3_^−^ reduction and NH_4_^+^ assimilation. As a result, total free amino acids and soluble proteins accumulated in leaves of *AMT1-3*-overexpressing plants, which depleted total organic acids to provide carbon skeletons for amino acid production. This large consume of organic acids promoted the breakdown of sugars. Conversely, the expression levels of *RUBISCO*, *PEPC1*, *PEPC2*, *PEPC3*, *PEPC4*, *PEPC6* and *PEPC7* dramatically increased in leaves of *AMT1-3*-overexpressing plants to produce more carbohydrates (Figure 7).

Plants have evolved a complex profile of responses to cope with changes in soil nitrogen availability, which is mediated by a stringent control of expression and/or activity of proteins involved in nitrogen transport and assimilation [59]. Recent studies on nutrient effects in plants have focused on their potential roles as signaling molecules in addition to their roles as building blocks of organic matter or cofactors [60]. Nitrogen sensing appears to regulate a variety of physiological and developmental processes in plants [8,61]. For example, NO_3_^−^ is a positive signal required for the induction of NO_3_^−^ uptake and its reduction, and the metabolized products of NO_3_^−^, NH_4_^+^ and its assimilation products Glu and Gln are believed to exert negative effects on NO_3_^−^ uptake and reduction [35,62,63,64,65]. Bacterial and fungal AMTs have been demonstrated to act as transceptors with dual functions as ammonium transporters and receptors that mediate ammonium-triggered changes in morphology or the transcription of target genes [66]. Plant AMTs may also be transceptors. For example, AMT1-3 can regulate lateral root branching in response to localized ammonium supplies [67]. More recent reports have demonstrated that *OsAMT1-3* is expressed specifically in roots but repressed by nitrogen, which indicates that *OsAMT1-3* participates not only in ammonium uptake but also in ammonium sensing in rice [14,35]. In this study, we expected to enhance ammonium uptake capacity in roots and to improve nitrogen fertilizer use efficiency and yield formation by overexpressing the *AMT1-3* gene in rice. In contrast, our transgenic plants grew poorly and displayed a low yield production and an unbalanced carbon and nitrogen metabolic status (Figure 3; Table 2 and Table 3). We hypothesized that the *AMT1-3* transcriptional level may act as a signal sensor to regulate carbon and nitrogen metabolism in rice. *AMT1-3* is a high-affinity NH_4_^+^ transporter gene, its transcriptional level is mainly expressed in rice roots under low nitrogen in the environment and repressed with nitrogen supplementation [13,35] The high transcriptional level of *AMT1-3* in our transgenic rice may mimic a nitrogen starvation signal in the environment and thus retard plant growth and development to ensure the plant can complete its entire life cycle. Recently, Gaur *et al.* validated *OsAMT1-3* as a biomarker for detecting available nitrogen pools both inside cells and in the soils around the root [14]. Similar results were reported by Yuan *et al.* who attempted to increase the ammonium uptake capacity via ectopic expression of *AMTs*; however, this largely failed because their transport capacities are tightly regulated by ammonium [31]. In addition, excess ammonium intake deregulates cellular pH homeostasis and primary metabolism [68]. Therefore, plant roots repress ammonium uptake at elevated ammonium supplementation [49,64]. Furthermore, several published works have demonstrated that *C*-terminal phosphorylation can mediate intermonomeric trans-inhibition of ammonium transport in plant roots and that the allosteric regulation of transporter activities even extends to heteromeric AMT protein complexes in *Arabidopsis* [47,49]. Additionally, *N*-terminal cysteines were demonstrated to affect oligomer stability of the allosterically regulated ammonium transporter LeAMT1-1 [50]. Thence, the post-transcriptional and post-translational regulation of plant AMT cannot be ignored in further studies.

## 4. Materials and Methods

### 4.1. Constructs and Transformation

“Ammonium transporter 1-3, rice” was used as the keywords to search the NCBI database (National Center for Biotechnology Information; Available online: http://www.ncbi.nlm.nih.gov) and the complete *OsAMT1-3* mRNA sequence (AF289479) was found. The full-length cDNA of *OsAMT1-3* gene was amplified by PCR from the rice (Zhonghua 11, *Japonica*) genome using the forward primer 5'-CATAGGACTCCTGTGTTGAGCGCGCGTCGA-3' and reverse primer 5'-CTGCCTGCAGGTACTACTAGCTCAGCTCCT-3', which contained the enzyme site of BamHI and PstI, respectively. Then the cDNA fragment was ligated into the pCAMBIA 1301S vector which driven by *CaMV* (Cauliflower Mosaic Virus) 35S promoter. All the procedures of DNA manipulation were according to the standard molecular techniques [69,70]. The chimeric gene was transformed into Zhonghua 11, a *japonica* rice cultivar, to obtain the *AMT1-3*-overexpressing transgenic plants by the *Agrobacterium tumefaciens*-mediated transformation method [71].

### 4.2. Plant Growth Conditions

Seeds of the wild type and *AMT1-3*-overexpressing rice germinated and were sowed in sand. The wild type and *AMT1-3*-overexpressing seedlings with four leaves were transferred into the experimental pots under the 0× N (fertilized with 0.15 g P_2_O_5_ and 0.2 g K per kg soil), 0.1× N (fertilized with 0.02 g·N, 0.15 g P_2_O_5_ and 0.2 g·K·per·kg soil), 1× N (fertilized with 0.2 g N, 0.15 g P_2_O_5_ and 0.2 g K per kg soil) and 5× N (fertilized with 1 g·N, 0.15 g·P_2_O_5_ and 0.2 g K·per kg·soil) conditions. The yield and its components were analyzed at the mature stage. In addition, the wild type and *AMT1-3*-overexpressing seedlings with four leaves were transplanted hydroponically under the 0× N (without NH_4_NO_3_), 0.1× N (with 1/10 NH_4_NO_3_), 1× N (normal nutrient solution described by Yoshida *et al.* [72]) and 5× N (with 5-fold NH_4_NO_3_) conditions. The culture solution was refreshed every 3 days. At the tillering stage and the heading stage, the roots, stems and leaves were harvested for the growth phenotype, leaf SPAD value and photosynthetic capacity analysis; for the carbon and nitrogen content, soluble proteins and carbohydrates concentrations determination; for the metabolic profiling and gene expression investigation.

### 4.3. Northern Blot, Southern Blot and Gene Expression Analysis

Leaf total RNA of the wild type and transgenic plants was extracted using TriZol reagent (Invitrogen, Karlsruhe, Germany), and 18 μg total RNA was used for Northern blotting to check whether the expression level of *OsAMT1-3* gene was accumulated in transgenic plants. Hybridizations were performed with a ^32^P-labeled probe, which is a partial specific fragment of *OsAMT1-3* gene. For Southern blot, genomic DNA was extracted from the leaves of transgenic plants by the CTAB method, and 4 μg of genomic DNA was digested by BamHI. The DNA was then transferred to a Hybond N+ nylon membrane (Amersham, Buchinghamshire, UK) for Southern blot analysis. Hybridizations were performed with a P32-labeled partial DNA fragment of the *hygromycin* gene. The results were detected by autoradiography. All the experimental procedures were according to the instructions described by Sambrook *et al.* [69] previously. For q-RT PCR assay, both roots and leaves of the wild type and *AMT1-3*-overexpressing plants were harvested from three biological replications under four different nitrogen conditions (0× N, 0.1× N, 1× N and 5× N) at the tillering stage. Total RNA was extracted using TriZol reagent (Invitrogen) and treated with DNaseI (Invitrogen) to avoid the genomic DNA contamination. The first-strand cDNAs were synthesized using Superscript III reverse transcriptase (Invitrogen). Table S2 listed the gene-specific and *actin* primers. Q-RT PCR assay was then performed in an ABI PRISM 7500 real-time PCR system (Applied Biosystems, Foster City, CA, USA).

### 4.4. Nitrogen Uptake Assay

Seeds of the wild type and *AMT1-3*-overexpressing rice germinated and were sowed in sand. At the 3-leaf stage, seedlings were transplanted hydroponically into the normal nutrient solution described by Yoshida *et al.* [72], except that 1.44 mM NH_4_NO_3_ was replaced by 2.88 mM NH_4_Cl. At the tillering stage, the NH_4_Cl in the nutrient solution was replaced with ^15^NH_4_Cl. Three biological replicated roots, stems and leaves were harvested at the time of 1, 3, 8 h, 1 and 3 days after ^15^N replacement. Then the ^15^N and total N content were analyzed by an isotope mass spectrometer (ANCA-MS, Europa Scientific, Crewe, UK) and by a C/N analyzer (Elementar, Vario MAX CN, Germany), respectively. Additionally, the total carbon and nitrogen content in roots, stems and leaves of wild type and *AMT1-3*-overexpressing plants under four different nitrogen conditions (0× N, 0.1× N, 1× N and 5× N) at the tillering stage were also determined by the C/N analyzer.

### 4.5. Physiological Parameters Determination

At the tillering stage, a chlorophyll meter (SPAD-502) was used to determine the SPAD value in flag leaves of the wild type and *AMT1-3*-overexpressing plants; a Li-6400XT portable photosynthesis system (Li-COR, Lincoln, NE, USA) was used to determine the photosynthetic parameters in flag leaves of the wild type and *AMT1-3*-overexpressing plants. Ten randomly selected plants were determined. The results were the average value of the upper, middle and bottom portion of each flag leaf. For soluble proteins analysis, three biological replicated roots, stems and leaves of the wild type and *AMT1-3*-overexpressing plants under four different nitrogen conditions (0× N, 0.1× N, 1× N and 5× N) were harvested at both the tillering and heading stages. Fresh samples were ground and homogenized with the Trizma extraction buffer described by Melo *et al.* [73] on ice. After centrifuging (12,000× *g*, 20 min, 4 °C), the supernatant was used to measure the soluble protein concentration based on the protocols of Bradford [74] protein assay with the bovine serum albumin as a standard protein. For soluble carbohydrates analysis, three biological replicated roots, stems and leaves of the wild type and *AMT1-3*-overexpressing plants under four different nitrogen conditions (0× N, 0.1× N, 1× N and 5× N) were harvested at both the tillering and heading stages. All the samples were dried and ground to powder. The soluble carbohydrates were extracted with boiling water and centrifuged at 12,000× *g* for 10 min. The supernatant was used to measure the soluble carbohydrate concentration according to the anthrone procedure [75,76]. For individual carbon and nitrogen metabolites analysis, three biological replicated roots and leaves of the wild type and *AMT1-3*-overexpressing plants under 0× N and 5× N conditions were harvested at the tillering stage. The individual metabolites involved in carbon and nitrogen metabolism were determined using GC-TOF-MS method from 50 mg fresh samples. The sample extraction, the data pre-treatment and normalization, the alignments and the metabolite identification were performed as described by Kusano *et al.* [77,78] and Redestig *et al.* [79].

## 5. Conclusions

In this study, we obtained the *AMT1-3*-overexpressing transgenic rice plants and systematically analyzed the effect of *AMT1-3* overexpressing on the rice growth, carbon-nitrogen metabolic status and gene expression profile at both the tillering and heading stages under four different nitrogen conditions (0× N, 0.1× N, 1× N and 5× N). Our results indicated that the overexpressing of *AMT1-3* gene caused a poor growth and yield in transgenic plants. The unbalanced carbon-nitrogen metabolic status might be the main reason. However, how *AMT1-3* overexpressing could lead to the carbon-nitrogen imbalance is still on going.

## Supplementary Materials

Supplementary materials can be found at http://www.mdpi.com/1422-0067/16/05/9037/s1.
