# Supplementary material for: Overexpressing of OsAMT1-3, a High Affinity Ammonium Transporter Gene, Modifies Rice Growth and Carbon-Nitrogen Metabolic Status
Source: Int J Mol Sci. 2015 Apr 23;16(5):9037–63. doi: 10.3390/ijms16059037 (PMC4463577; doi:10.3390/ijms16059037)
Supplement: Supplementary file 1 [file ijms-16-09037-s001.pdf]

Supplementary Information

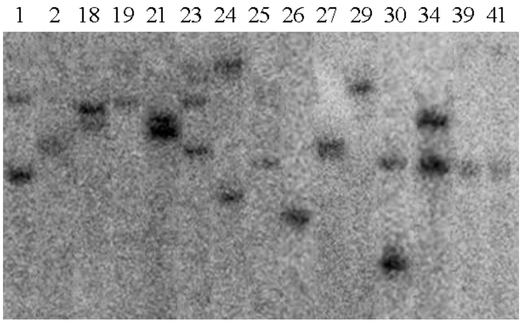

Figure S1. Southern blot analysis of *AMT1-3*-overexpressing plants.

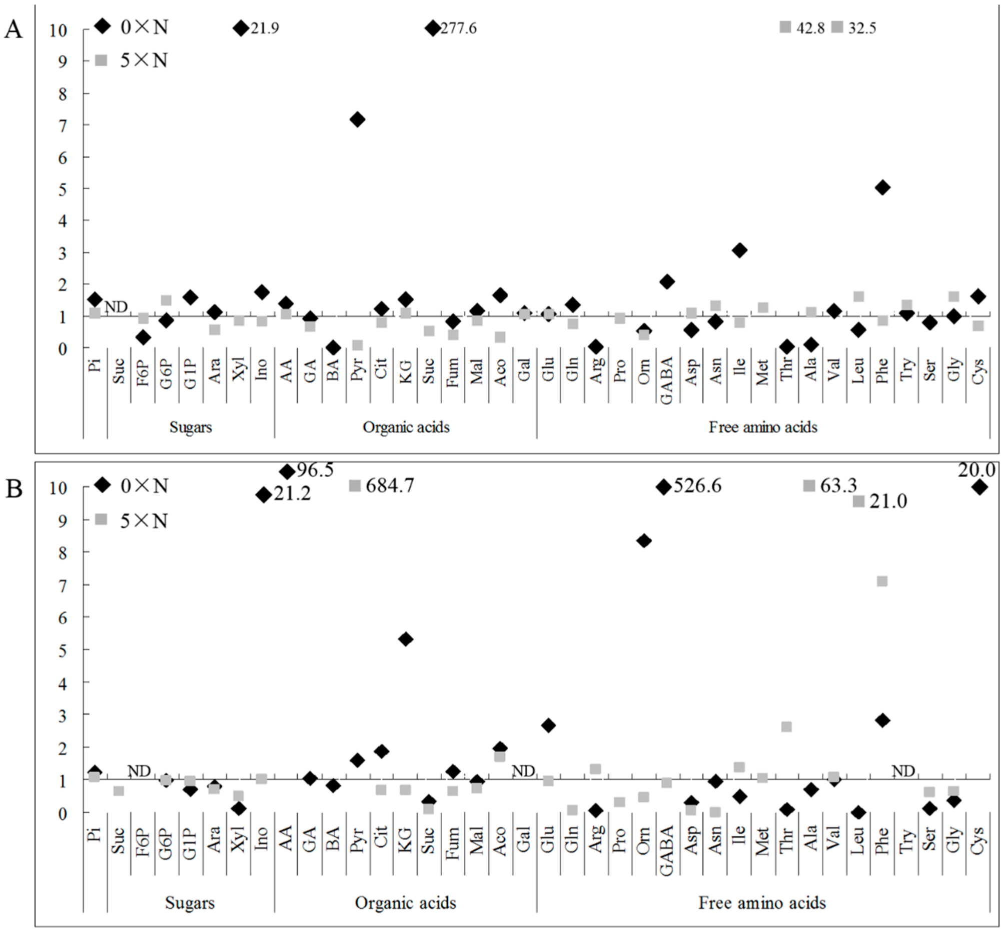

Figure S2. Fold change corresponds to the ratio of the concentration of individual metabolites involved in carbon and nitrogen metabolism in the *AMT1-3*-overexpressing plants relative to the wild type plants for the leaves (**A**) and roots (**B**) at the tillering stage. Pi, phosphate; Suc (in the group of sugars), sucrose; Fru, Fructose; F6P, Frutose-6-P; G6P, Glucose-6-P; G1P, Glucose-1-P; Ara, Arabinose; Xyl, Xylitol; Ino, Inositol; AA, Ascorbic acid; GA, Glutaric acid; BA, Benzoic acid; Pyr, Pyruvate; Cit, Citrate; KG, Ketoglutarate; Suc (in the group of organic acids), Succinate; Fum, Fumarate; Mal, Malate; Aco, Aconitase; Gal, galactose; Glu, Glutamate; Gln, Glutamine; Arg, Arginine; Pro, Proline; Orn, Ornithine; GABA, Aminobutyric; Asp, Aspartate; Asn, Asparagine; Ile, Isoleucine; Met, Methionine; Thr, Threonine; Ala, Alanine; Val, Valine; Leu, Leucine; Phe, Phenylalanine; Try, Tryptophan; Ser, Serine; Gly, Glycine; Cys, Cysteine.

Table S1. Fold change corresponding to the ratio of the gene expression level in the *AMT1-3*-overexpressing plants relative to the wild type plants for the roots and leaves at the tillering stage under 0× N, 0.1× N, 1× N, 5× N conditions.

	**0× N**		**0.1× N**		**1× N**		**5× N**	
	**Root**	**Leaf**	**Root**	**Leaf**	**Root**	**Leaf**	**Root**	**Leaf**
NRT1;1	0.44 **	0.46 **	1.66	2.36 **	3.53 **	1.00	3.10 **	3.87 **
NRT1;2	0.88	1.09	2.52 **	2.12 *	1.39	0.64 **	1.32 **	1.59
NRT2	2.35 **	1.71 *	0.59 **	1.46 *	0.71 *	3.65 **	0.53 **	1.67
NR1	1.96 **	0.36 **	0.01 **	0.21 **	0.96	0.14 **	2.01 **	2.21 **
NR2	0.79	0.23 **	0.21 *	2.28 **	1.41 *	0.66 *	1.79 **	1.17 *
GS1;1	0.48 **	0.74 *	1.16	1.03	0.97	0.79	2.50 **	1.43 *
GS1;2	1.12	0.82	2.36 **	1.52 **	0.69 *	1.08	0.90	2.49 **
GS1;3	0.76	0.84 *	3.89 **	7.47 **	2.50 **	1.22	1.94 **	1.78 **
GS2	0.56 **	0.55 **	2.46 **	1.63 **	1.77 *	1.20	0.73 *	2.39 **
Fd-GOGAT1	0.49 **	1.03	0.49 **	1.40 **	1.56 **	1.02	1.62 **	1.62 **
Fd-GOGAT2	1.12	0.53 **	1.74 **	1.06	1.53 *	1.26 **	1.30 *	1.52 **
NADH-GOGAT1	1.24 **	0.77 **	0.48 **	1.73 **	1.46 **	0.88	1.07	2.29 **
NADH-GOGAT2	0.74	1.85 **	0.24 **	2.96 **	0.65 **	2.36 **	0.19 **	1.86 **
RUBISCO	1.05	0.37 **	0.25 **	1.53 **	2.48 **	1.73 **	1.78 **	2.20 **
PEPC1	0.63 **	0.55 **	1.11	2.13 **	0.23 **	0.75	3.62 **	2.17 **
PEPC2	0.15 **	0.57 **	1.22	2.34 **	1.37 *	1.44 *	0.43 **	1.85 **
PEPC3	0.25 **	0.83 *	0.62 **	0.98	0.57 *	1.36 *	3.03 **	3.77 **
PEPC4	0.40 **	0.98	0.48 **	1.48 **	1.37	1.48 **	2.90 **	3.05 **
PEPC6	0.98	1.02	1.46 **	0.97	2.18 **	0.88	3.91 **	2.54 **
PEPC7	0.84	0.75 *	1.61 **	1.32 **	0.49 *	1.43 **	2.14 **	1.82 **

*, ** indicate the significant differences between the *AMT1-3*-overexpressing plants and wild type plants at the level of p = 0.05 and p = 0.01, respectively, from three biological replications. NRT: nitrate transporter; NR: nitrate reductase; GS: glutamine synthetase; GOGAT: glutamate synthase; RUBISCO: Ribulose-1,5-bisphosphate carboxylase/oxygenase; PEPC: phosphoenolpyruvate carboxylase.

Table S2. Primer sequences of the key genes involved in the carbon and nitrogen metabolism used in qRT-PCR.

Gene Name	cDNA Accession NO.	Primer Sequence
NRT1;1	AK066920	F: CCTCGCAAGTGACCCTTGAAT<br>R: CGATGGCTAATGAGGAACCCTT
NRT1;2	AK101480	F: GAACATGCGGATCATGTCGTT<br>R: CGATCACGGAGCTGTACATGAG
NRT2	AK109733	F: TTCGCGAACCCGCATATGA<br>R: GTTGAGGTTGTCGCGGATGAT
NR1	AK102178	F: ACTACCATTACCGCGACAACC<br>R: CTCGTTTATCATGTACTCCGGC
NR2	AK121810	F: AGCTGAACGTGAACTCGGTGA<br>R: AGGCGTATCCCTTCATGGTGT
GS1;1	AK109397	F: GAGTCGTCGTCTCATTTGACCC<br>R: GTAGCCACCATCGTTCCTCATC
GS1;2	AK243037	F: TTTTCAAGGACCCGTTCAGGA<br>R: CGGCACTGTGCCTCTTGTTAGT
GS1;3	AK099290	F: TCAAGCCATCTTCAGAGACCCA<br>R: TACCGGTTGTTCGTCGGAATC
GS2	AK063706	F: AGGATCGGACAAATCGTTTGG<br>R: GCATGACCTCTCCATTTGTTCC
Fd-GOGAT1	AK102025	F: AAATGCCTCTTTGCAAGGCC<br>R: GACTGTGAG CCCCATCCAAATA
Fd-GOGAT2	AK068130	F: CCGATGCGATTGAGAATGAGA<br>R: CTTCTTGGCAATGACACCTGC
NADH-GOGAT1	AK105755	F: TGCTTGAGAGAATGGCGCA<br>R: AACCCAGCATCCTTTGTCACC
NADH-GOGAT2	AK070485	F: GGTTGTCATTGGTGGTGGAGA<br>R: TGGTGGCTCTGGCAAAAGTT
RUBISCO	AK243615	F: AGGCTTCAAATTGCCGTTGA<br>R: TCTAGGCCATCCAGTTCCTCCT
PEPC1	AK100688	F: ACATTCCGTGTTGCTGCAGAG<br>R: TGCAACAGTTCAACCGCTAGG
PEPC2	AK066635	F: CAGAAGCACGCAAGCATTAGG<br>R: CGCGAGAATCTCTCTCTGAAGG
PEPC3	AK101274	F: ACCGGTCCATTGTCTTCCAAG<br>R: CGTTTTGATGGCCTACTTCCAA
PEPC4	AK065425	F: TGGATGAGATGGCTGTTGTGG<br>R: TTCTGTCTCAGGTGTTGCCGA
PEPC6	AK073703	F: ATGTCTGCCAGGCTTACACGAT<br>R: CGGCTTAGACCAGTCCATGATC
PEPC7	AK242583	F: GAGTATTTCCGCCTTGCAACAC<br>R: ACGGAGTGATTCAATGCCTCC
ACTIN	AK070531	F: GACAATGGAACCGGAATGGTC<br>R: CCCAACCATAACGCCTGTATGT
